# Congruence between patients’ preferred and perceived participation in medical decision-making: a review of the literature

**DOI:** 10.1186/1472-6947-14-25

**Published:** 2014-04-03

**Authors:** Linda Brom, Wendy Hopmans, H Roeline W Pasman, Danielle RM Timmermans, Guy AM Widdershoven, Bregje D Onwuteaka-Philipsen

**Affiliations:** 1 Department of Public and Occupational Health, EMGO Institute for Health and care research, VU University Medical Center, Van der Boechorststraat 7, 1081 Amsterdam, BT, The Netherlands; 2 Department of Medical Humanities, EMGO Institute for Health and care research, VU University Medical Center, Amsterdam, The Netherlands

**Keywords:** Patient participation, Decision-making, Patient involvement, Congruence, Review

## Abstract

**Background:**

Patients are increasingly expected and asked to be involved in health care decisions. In this decision-making process, preferences for participation are important. In this systematic review we aim to provide an overview the literature related to the congruence between patients’ preferences and their perceived participation in medical decision-making. We also explore the direction of mismatched and outline factors associated with congruence.

**Methods:**

A systematic review was performed on patient participation in medical decision-making. Medline, PsycINFO, CINAHL, EMBASE and the Cochrane Library databases up to September 2012, were searched and all studies were rigorously critically appraised. In total 44 papers were included, they sampled contained 52 different patient samples.

**Results:**

Mean of congruence between preference for and perceived participation in decision-making was 60% (49 and 70 representing 25^th^ and 75^th^ percentiles). If no congruence was found, of 36 patient samples most patients preferred more involvement and of 9 patient samples most patients preferred less involvement. Factors associated with preferences the most investigated were age and educational level. Younger patients preferred more often an active or shared role as did higher educated patients.

**Conclusion:**

This review suggests that a similar approach to all patients is not likely to meet patients’ wishes, since preferences for participation vary among patients. Health care professionals should be sensitive to patients individual preferences and communicate about patients’ participation wishes on a regular basis during their illness trajectory.

## Background

Making medical treatment decisions can be difficult for patients and physicians. Both patients and physicians are increasingly expected to possess communication skills that facilitate patient participation [1,2]. It has also been argued that patients often need to be more assertive and involved to enable a patient-centred approach to medical decision-making [3,4]. In order to place the patient at the centre of care, it is expected that physicians perform their role in a less authoritarian manner [5]. A survey among physicians from different medical disciplines showed that they generally tended to be quite open towards patient participation [6]. However, research among patients shows that the majority do not always feel that their level of participation in medical decision-making is sufficient [7].

Preferences for participation in medical decision-making are often measured with tools such as the Control Preferences Scale (CPS) [8], evidence suggest the CPS is clinically relevant, easily administered and valid in health care decision-making [8]. The scale has been used in a variety of populations and settings, and administered in several ways, including written, verbal, using answering cards, or using only a 5-point scale.

Two systematic reviews addressed the issue of preferences for participation in medical decision-making. Chewning et al. [9] included various patient populations and reported a preference for shared or active decision-making, and noted that this preference for participation increased over the past three decades. Tariman et al. [10] reviewed studies on patients with different types of cancer that compared their preferred and perceived role during decision-making. All studies in this review showed discrepancies between preferred and perceived roles in decision-making, and in the majority of the studies patients preferred to be more involved in decision-making than perceived.

Additionally, several original studies have reported on factors associated with preferred role in medical decision-making. Studies have shown that younger patients, higher educated people and women more frequently prefer a more active role in decision-making [11,12]. In contrast, older people were found to more often favour a more ‘paternalistic attitude’ from their physician [13]. The phase in the illness trajectory also appears to influence patient preferences regarding participation. For example, a study among patients with prostate cancer showed that they preferred a more active role later in their disease, possibly because of getting used to being ill [14].

We performed a review including any study population with a strong focus on the congruence between a patient’s preferred and perceived role in decision-making. We also investigated factors associated with preferences, perceived participation and the congruence between these two. Of importance when exploring this issue, we distinguished between prospective and retrospective measurement of preferences. In some studies preferences are assessed prospectively, that is before decision-making takes place, while in other studies the patient preferences are measured retrospectively, that is after decision-making took place. When measured retrospectively patients probably take into account their evaluation of the decision-making when expressing their preference after the decision-making took place, while prospectively measured preferences are not biased by knowledge of the actual decision-making process. However, prospective measures cannot provide insight in changes in preferences that might occur during the decision-making process or over time [14,15].

The aim of our study was to give an overview of the results from studies in which congruence between patients’ preferred and their perceived participation in medical decision-making is assessed and when no congruence was measured, if patients preferred more or less participation than perceived. We distinguished prospective and retrospective studies and also extracted data on factors associated with congruence. Knowing more about presence of congruence and (direction of) mismatch in medical decision making is relevant for practice, since a lack of knowledge may result in sub optimal care and serve as a barrier to patient-centred care.

## Methods

### Search strategy

A systematic review of the research literature was carried out to identify studies that examined the congruence between preferred and perceived participation in medical decision-making among patients. To identify relevant studies, we searched Medline, PsycINFO, CINAHL, EMBASE and the Cochrane Library databases up to September 2012. A “medical information specialist” from the Medical Library VU University medical center assisted with development and pilot testing of the search strategy. The following search terms were used, *preference, patient participation* and *decision-making*.

### Selection criteria

All inclusion and exclusion criteria were established before conducting the database searches. Studies were included if they met the following criteria: (a) reported patients’ preferences and/or patients’ perceived participation and the congruence between preferred and perceived patient participation (or this could be calculated from the presented data), (b) used a 3- or 5-point scale measuring involvement of patient participation in decision-making (active, shared, passive) (not necessarily the CPS specifically) and (c) concerned medical treatment decision-making. All study types could be included.

Studies were excluded when they concerned preferences for participation in medical decision-making as a surrogate (e.g. in children) and when the manuscript was not written in English.

### Study identification and data extraction

Articles were entered into Reference Manager 11. Titles and abstracts were initially screened by two reviewers independently (LB and WH) to decide if the full text articles should be obtained. When there was disagreement, this was resolved through discussion with a third reviewer (HRWP). Full text articles were excluded if a more detailed examination showed that papers did not fit the inclusion criteria.

Two pairs of reviewers (LB and BDOP, WH and HRWP) determined final inclusion or exclusion using a standardized checklist of items. We created a checklist including an inventory of the features critical to inclusion and assessment of the study: 1) type of illness, 2) type of treatment decision, 3) prospective or retrospective measurement of preferences, 4) percentage of preferred and/or perceived participation, 5) percentage of congruence and direction of mismatch (preferred more, or preferred less participation than perceived) and 6) associations with socio-demographic factors. We piloted this checklist in four articles for completeness and reliability reviewers. This pilot resulted in no major additions or modification to items in the original checklist. When analysing the articles with the checklist, discrepancies between the two reviewers were resolved by consensus and if necessary consultation with a third reviewer.

Patients’ preferred and/or perceived participation in the included studies were described in three categories: active, shared or passive. If data was presented on a 5-point scale (A,B,C,D,E), we rearranged it into a 3-point scale (AB, C, DE) to create uniformity. A large number of studies measured on a 3-point scale (e.g., mainly the patient, patient and provider equally, mainly the provider) [12,15-22]. Congruence, mismatch and its direction were reported by using data presented in the original article (based on a 3-point scale) or calculated based on the numbers reported for preferred and perceived participation. Congruence was calculated then by counting the number of participants whose preferred and perceived participation was equal and divided it by the total number of participants. Direction of mismatch was calculated by counting number of patients whose preferred participation was different from perceived participation (preferred more, or preferred less participation than perceived) divided it by the total number of participants.

This resulted in three categories, namely: 1) congruent, 2) preferred more participation than perceived or 3) preferred less participation than perceived.

If more than one study sample was described in an included article, data was extracted for the reported samples separately, for example the different groups in randomised controlled trials or when two patient samples were compared within an included article.

We divided the studies and reported the findings separately for preferences and congruence with perceived participation as follows a) prospective vs retrospective studies, b) patients with cancer vs patients without cancer (non-cancer), and c) treatment options asked in general vs specific treatment options.

The Mixed Methods Appraisal Tool (MMAT) was used to appraise the methodological quality of the included studies [23]. The MMAT has been designed for a systematic mixed studies review. The scores range from 0 (no criteria met) to 4 (all criteria met).

## Results

### Study selection

**Figure 1:**
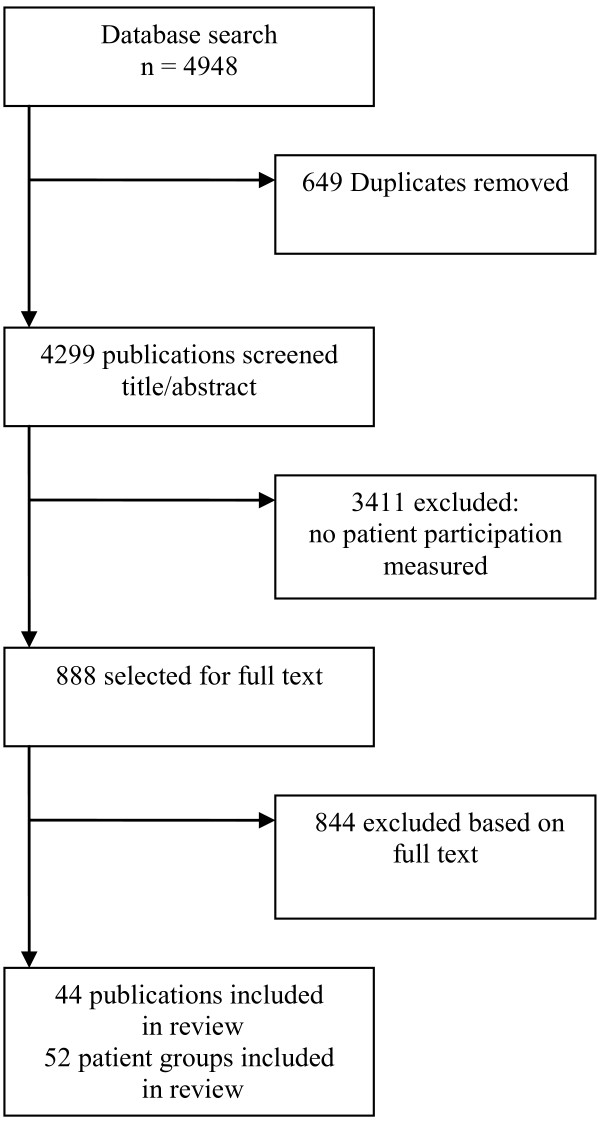
Flowchart of the literature search.

The results of the search strategy are shown in Figure 1. A total of 4299 unique citations were identified. The abstracts were reviewed and 888 articles were selected for a full-text review. The most important reason for exclusion on the basis of the abstract was that studies did not report patient participation in a medical decision-making situation, most evaluated outcomes of treatments. A further included 844 studies were excluded after full-text review as they did not report on congruence between patients’ preferences and perceived participation. A total of 44 papers were included which contained 52 different patient samples: A total of 12 studies in which preference was measured prospectively (Table 1) reported on 15 patient samples (3 RCTs with intervention and control groups). Thirty-two studies in which preference was measured retrospectively (Table 2) were included. One RCT with an intervention group and a control group, and 2 studies comparing patient populations with different types of cancer, resulted in a total of 37 different patient samples.

**Table 1:** Patients’ preferences, congruence and direction of mismatch in medical decision-making

		**Congruence between preferred and perceived participation %**			**Preferences in participation %**		
		**Yes, preferred and perceived participation were equal**	**No, preferred more participation**	**No, preferred less participation**	**Active**	**Shared**	**Passive**
Leighl, 2011 Canada [24]		32	30	38	20	41	39
Brown, 2012, USA [15]		37	36	28	24	48	28
Ramfelt, 2000 Sweden [25]		44	48	8	6	62	32
Janz, 2004 USA [26]		46	17	36	39	48	13
Gattellari, 2001 Australia [27]		48	29	23	11	50	39
Wunderlich, 2010 USA [28]		49	19	32	13	21	66
Butow, 2004 Australia [29]							
Control group		57	13	29	13	52	35
Intervention group		50	31	18	11	50	39
Kasper, 2008 Germany [30]							
Control group		55	23	22	65	24	11
Intervention group		52	33	15	78	13	9
Degner, 1997 Canada [12]		55	32	13	22	44	34
Ernst, 2010 Germany [17]		56	23	21	12	28	60
Davison and Degner, 2002 Canada [31]							
Control group		66	25	9	34	50	16
Intervention group		80	13	7	28	49	34
Clayton, 2011 USA [32]*		90	-	-	34	33	33
**Total**	Mean	53	29	21	28	44	29
Percentiles 25^th^ - 75^th^		45-56	19-36	13-29	14-34	35-50	14-39

**Preference prospectively measured** (n = 12 studies, n = 15 patient samples).

Note *: studies reported% of congruence, but not% for direction of mismatch.

Table sorted on% congruence.

**Table 2:** Patients’ preferences, congruence and direction of mismatch in medical decision-making

		**Congruence between preferred and perceived participation %**			**Preferences in participation %**		
		**Yes preferred and perceived participation were equal**	**No, preferred more participation**	**No, preferred less participation**	**Active**	**Shared**	**Passive**
Ramfelt et al., 2005 Sweden [33]		31	55	14	18	47	35
Caress, 1997 UK [34]		36	54	10	49	31	16
Sepucha et al., 2009 USA [22]		38	46	16	8	88	4
Hack et al., 2006 Canada [35]		48	41	12	36	42	23
Kremer et al., 2007 USA [36]		49	25	25	28	59	13
Bilodeau et al., 1996 Canada [37]*		50	-	-	20	37	43
Caress et al., 2005 UK		51	43	6	24	36	40
Beaver and Booth, 1999 UK [38]							
Colorectal cancer		52	33	16	4	17	78
Breast cancer		60	22	18	20	28	52
Purbrick et al., 2006 UK [39]		56	18	26	10	69	21
Carey et al., 2012 Australia [40]		56	34	10	26	30	45
Jefford et al., 2011 Australia [41]		57	27	12	29	37	30
Nakashima et al., 2012 Japan [42]		59	24	16	18	69	13
Ford et al., 2003 UK [43]		60	25	15	18	46	36
Caldon, 2008 UK [44]		61	9	29	40	42	17
Krist et al., 2007 USA [45]**							
Control group		61	23	16	-	-	-
Brochure group		69	21	10	-	-	-
Website group		73	16	11	-	-	-
Vogel et al., 2008 Germany [46]		63	22	15	31	29	40
Wallberg et al., 2009 Sweden [47]		66	27	7	10	68	23
Beaver et al., 2007 UK [48]*							
Gyneacolocal cancer		66	23	11	21	32	47
Breast cancer		39	-	-	15	24	61
Colorectal cancer		61	-	-	7	13	80
Hawley et al., 2007 USA [49]**		66	13	21	-	-	-
Zhang et al., 2011 China [50]		69	28	3	11	46	44
Mahone, 2008 USA [19]*		69	-	-	11	82	17
Lantz et al., 2005 USA [51]**		69	11	20	-	-	-
Murray et al., 2007 UK [20]		71	17	12	28	62	9
Pardon et al., 2011 Belgium [52]		71	20	9	15	22	63
Davidson et al., 1999 Canada [53]		71	29	0	19	24	57
Wallberg et al., 2000 Sweden [54]		72	20	8	13	21	66
Joliceur et al., 2009 Canada [55]		77	23	0	15	54	31
Lam et al., 2003 China [18]		80	7	13	33	59	8
Mohamedali et al., 2010 Canada [56]*		80	-	-	11	52	37
Vogel et al., 2009 Germany [57]**		84	2	15	-	-	-
Hawley et al., 2008 USA [58]**		93	4	3	-	-	-
Palmer et al., 2012 USA [21]		97	3	1	45	39	16
**Total**	Mean	63	24	13	21	43	36
Percentiles 25 - 75		54-71	16-29	8-16	11-28	29-59	17-48

**Preference retrospectively measured** (n = 32 studies, n = 37 patient samples).

Note *: Studies reported% of congruence, but not% for direction of mismatch.

Note **: Studies reported perceived participation.

Table sorted on% congruence.

### Study characteristics

Characteristics of the 12 prospective studies (15 patient samples) are shown in Table 3. A total of 3416 women and 645 men were included in these studies. Mean age was 56.3 years with a range from 21 to 89 years old.

**Table 3:** Study characteristics of 12 included prospective studies

	**Study population**			**Treatment decision**	**Study design**	Quality of studies**
	**Number (gender)**	**Age (mean) (range)**	**Patient population**		**Data collection**	
**Preference prospectively measured**						
Brown et al., 2012 USA [15]	683 women	55.3	Breast cancer	General	RCT questionnaire before and after consultation	3
		-				
Butow et al., 2004 Australia [29]	75 men	58	Cancer	General	RCT Questionnaires before and after consultation	3
	89 women	-				
Clayton et al., 2011 USA [32]	62 men	43.2	Patients from general practice	General	self-reported data	2
	107 women	18-89				
Davison and Degner, 2002 Canada [31]	749 women	58.3	Breast cancer	General	Prospective, blocked, two-arm randomized controlled trial	1
		-				
Degner et al., 1997 Canada [12]	1012 women	-	Breast cancer	Operation	Cross-sectional, consecutive sampling, nurse administrated questionnaire	3
		58.3				
Ernst et al., 2010 Germany [17]	59 men	57	Hemato-oncological illnesses	General	Postal review interview	1
	45 women	21-84				
Gattellari et al., 2001 Australia [27]	133 men	56.7	Cancer	General	RCT prospective, cross-sectional consecutive sampling self-administered questionnaire	4
	100 women	22-82				
Janz et al., 2004 USA [26]	101 women	54.9	Breast cancer	Operation	Telephone interviews	4
		34-81				
Kasper et al., 2008 Germany [30]	79 men	43.1	MS	Medication	RCT; telephone and post, standardized questionnaires	4
	218 women	-				
Leighl et al., 2011 Canada [24]	62 men	62.5	Colorectal cancer	Medication	RCT	2
	38 women	-				
Ramfelt et al., 2000 Sweden [25]	41 men	70	Colorectal cancer	Operation	Questionnaires	3
	45 women	34-84				
Wunderlich et al., 2010 USA [28]	134 men	58	Colorectal cancer	Screening	Pre- and post-visit survey	1
	229 women	-				

Table sorted alphabetically.

Note **:Methodological quality of studies assessed with MMAT(23), ranging from 0 (lowest) to 4 (highest).

Ten out of 12 studies included patients with cancer. Breast and colorectal cancer were the most common. In six studies preference for decision-making referred to a general preferences and in six studies preferences related to specific treatment options; operation (3 studies), medication (2 studies), screening (1 study).

Characteristics of the 32 retrospective studies (including 37 patient samples) are illustrated in Table 4. A total of 6088 women and 1709 men are represented in these studies (one study did not report gender, but included 2761 patients in total). Mean age was 56,4 years with an age range from 19 to 89 years old. Twenty-six out of 32 studies included patients with cancer. Breast cancer was the most common (14 studies). In 18 studies preference for treatment decision-making referred to general preference and in 14 studies preferences related specific treatment options; operation (9 studies), medication (3 studies), operation vs. medication (1 study), screening (1 study).

**Table 4:** Study characteristics 32 included retrospective studies

	**Study population**			**Treatment decision**	**Study design**	Quality of studies**
	**Number (gender)**	**Age (mean) (range)**	**Patient population**		**Data collection**	
**Preference retrospectively measured**						
Beaver et al., 1999 UK [48]	35 Men	66.6	Colorectal cancer	General	Cross-sectional, convenience sampling structured interview schedule	2
	13 Women	23-83				
Beaver and Booth, 2007 UK [38]	53 Women	55	Gynaecological cancers breast cancer colorectal cancer	General	structured interviews, consecutive sample	2
		24-82				
Bilodeau and Degner, 1996 Canada [37]	74 Women	-	Breast cancer	Operation	Cross-sectional, convenience sampling survey, Interview schedule	2
		18-83				
Caldon, 2008 UK [44]	356 Women	58.5	Breast cancer	Operation	Cross-sectional, convenience sampling questionnaire survey	2
		30-89				
Caress, 1997 UK [34]	245 Men	47.2	Breast cancer	General	Cross-sectional	3
	160 Women	16-82				
Caress et al., 2005 UK [40]	97 Men	51.9	Asthma	General	Cross sectional survey , structured interviews	2
	133 Women	19-94				
Carey et al., 2012 Australia [16]	158 Men	59.5	Haemato-logical cancer	General	Cross-sectional design, questionnaire	2
	110 Women	-				
Davidson et al., 1999 Canada [53]	10 Men	65	Lung cancer	General	Interview	2
	11 Women	-				
Ford et al., 2003 UK [43]	56 Men	49	Patients from general practice	General	Questionnaire	3
	115 Women	16-88				
Hack et al., 2006 Canada [35]	205 Women	59.5	Breast cancer	Operation	Interviews	2
		-				
Hawley et al., 2007 USA [49]*	1101 Women	59	Breast cancer	Operation	A self-administered survey of a population-based sample	3
		29-79				
Hawley et al., 2008 USA [58]*	877 Women	59	Breast cancer	Operation	Survey, data questionnaire	3
		29-79				
Jefford et al., 2011 Australia [41]	68 Men	58.4	Cancer	General	Convenience sample, questionnaires	3
	34 Women	29-85				
Joliceur et al., 2009 Canada [55]	13 Women	57	Ovarian cancer	General	Retrospective cross-sectional design. Face to face interviews (semi-structured)	2
		46-77				
Kremer et al., 2007 USA [36]	51 Men	42	HIV	Medication	Cross-sectional study	2
	28 Women	-				
Krist et al., 2007 USA [45]	497 Men	57	Prostate cancer	Screening	RCT	4
		50-70				
Lam et al., 2003 China [18]	154 Women	59	Breast cancer	Operation	Face-to-face interviews	2
		28-79				
Lantz et al., 2005 USA* [51]	1633 Women	-	Breast cancer	Operation	Cross-sectional, mailed survey	3
		-				
Mahone, 2008 USA [19]	49 Men	43	Serious mental illness	Medication	Cross-sectional, correlational study	1
	35 Women	20-62				
Mohamedali et al., 2010 Canada [56]	18 Men	-	AML	General	Questionnaires	4
	17 Women					
Murray et al., 2007 UK [20]	2761	-	American Public	General	Cross-sectional telephone survey	2
		-				
Nakashima et al., 2012 Japan [42]	104 Women	-	Breast cancer	General	Cross-sectional design, questionnaires	2
		-				
Palmer et al., 2012 USA [21]	181 Men	61.3	Prostate cancer	General	Cross-sectional case–control study, interview	3
		43-75				
Pardon et al., 2011 Belgium [52]	102 Men	-	Lung cancer	General	Questionnaire	4
	26 Women	64.4				
Purbrick et al., 2006, UK [39]	12 Men	63	Ocular cancer	Operation	Questionnaire	0
	27 Women	19-80				
Ramfelt et al., 2005 Sweden [33]	26 Men	69	Rectal cancer	Operation	Prospective, cross-sectional convenience sampling	2
	29 Women	34-83				
Sepucha et al., 2009 USA [22]	32 Women	55	Breast cancer	General	Patient survey	3
		37-78				
Vogel et al., 2008 Germany [46]	137 Women	53.75	Breast cancer	General	Self-explanatory questionnaire	3
		19-75				
Vogel et al., 2009 Germany* [57]	135 Women	54	Breast cancer	Mastectomy vs breast-concerving therapy vs chemotherapy	Consecutive sample self-administered questionnaire	3
		19-75				
Wallberg et al., 2000 Sweden [54]	201 Women	-	Breast cancer	General	Interviews	4
		-				
Wallberg et al., 2009 Sweden [47]	201 Women	60.7	Breast cancer	Medication	Questionnaires	1
		55.3-				
Zhang et al., 2011 China [50]	104 Men	45	Chronic hepatitis	General	Cross-sectional, mailed survey	3
	74 Women	18-69				

Note *:Studies reported perceived participation.

Note **:Methodological quality of studies assessed with MMAT (23), ranging from 0 (lowest) to 4 (highest).

*Table sorted alphabetically*.

In total, 38 studies used the Control Preferences Scale to assess patient decision-making preferences and perceived participation; preferences were measured in various ways (questionnaire, face to face interview, telephone survey).

Most included studies were conducted in Europe (8 UK, 4 Germany, 4 Sweden, 4 Germany, 1 Belgium) followed by the United States of America (12 studies) and Canada (8 studies) and Australia (4 studies). Only 3 studies were conducted in Asia (2 studies in China, 1 in Japan) (Tables 3 and 4).

Five included studies were published before the year 2000, 11 between 2000 and 2005 and the majority (28 studies) after the year 2005. Furthermore, 31 out of 44 studies had a moderate score (2 or 3 out of 4) for the methodological quality assessed by the MMAT (23), 7 studies (including 3 RCTs) met all 4 criteria for methodological quality, and 6 studies had a low score (0 or 1) for the methodological quality.

### Preferences

#### Prospective vs. retrospective

In many studies, most patients preferred a shared role in the decision-making process (in 10 out of 15 patient samples of prospective studies and in 16 out of 37 patient samples of retrospective studies). In retrospective studies, the majority of patients preferred a passive role more often than in the prospective studies (in 12 out of 37 retrospective patient samples and 2 out of 15 prospective patient samples) (Tables 1 and 2).

#### Cancer vs. non-cancer

Overall, patients with cancer more often desired a passive role than patients without cancer (14 out of 43 patient samples with cancer vs. zero out of six patient samples with non-cancer).

#### General vs. specific treatment options

When preferences were asked in general patients often desired a passive role (12 out of 29 patient samples). If preferences related to a specific treatment option were assessed(e.g., for example breast cancer surgery) patients less often desired a passive role (2 out of 22 patient samples).

### Congruence

All studies showed discrepancies between preferred and perceived roles in medical decision-making, shown in Table 1 and Table 2. The overall mean of congruence (all studies) was 60% (49 and 70 representing the 25^th^ and 75^th^ percentiles, respectively).

#### Prospective vs. retrospective

The percentages of congruence was slightly higher in retrospective studies than in prospective studies (mean 63% vs. mean 53%).

Where no congruence was found, in 36 patient samples most patients preferred more participation than perceived. In the prospective studies, 11 out of 15 patient samples preferred more participation than perceived and in the retrospective studies, 25 out of 33.

In 9 patient samples most patients preferred less participation than perceived. In the prospective studies, 3 out of 15 patient samples preferred less participation and in the retrospective studies, 6 out of 37. The latter all involved patients with cancer [18,26,28,29,39,44,49,51],[57].

#### Cancer vs. non-cancer

Congruence was similar for patients with cancer and for patients with non-cancer (60% and 59%, respectively).

#### General vs. specific treatment options

There was no difference in congruence between treatment preference asked in general and for specific treatment options (58% and 61%, respectively).

### Associations

Table 5 summarizes the factors associated with patient participation in medical decision-making, distinguishing associations with preferred role, perceived role and congruence.

**Table 5:** Reported significant associations with preferred role, perceived role and congruence

**Determinant**	**Associations studied for**	**Outcome**
Age (N = 16)	Preferred role (n = 13)	- Older prefer more passive role [20,35,40,53,54]
		- Younger prefer a more active role [12,35,44,50]
		- Younger prefer more often shared role [18,35,54]
		- No association found [33,37,38]
	Perceived role (n = 3)	- Older women perceived a more passive role [37]
		- Younger perceived less active [49]
		- No association found [44]
	Congruence (n = 1)	- No association found [17]
Gender (N = 1)	Congruence (n = 1)	- No association found [17]
Education level (N = 10)	Preferred role (n = 7)	- Higher educated prefer more often active role [12,20,26,42,54]
		- Lower educated patients prefer more often passive role [40,50]
	Perceived role (n = 1)	- No association found [35]
	Congruence (n = 2)	- If high school or less; patients preferred less involvement [49]
		- No association found [17]
Socioeconomic status (N = 1)	Preferred role (n = 1)	- Higher income prefer more active [22]
Ethnicity (N = 2)	Preferred role (n = 1)	- Black patients (vs. white) prefer more passive [20]
	Perceived role (n = 1)	- Latina-Spanish speaking women preferred more involvement [58]
Marital status (N = 3)	Preferred role (n = 3)	- If partner than more often preference for a shared or passive role [36]
		- Widowed more like to prefer passive role [35]
		- Married, (who had lumpectomy and whose first language was English) prefer more active/shared roles [12]
QoL (N = 2)	Congruence (n = 2)	- No association found [52,57]
Depression/Anxiety (N = 3)	Preference (n = 1)	- Patients who preferred a passive role were more depressed [46]
	Congruence (n = 2)	- Lower depression scores if congruence [57]
		- Mismatch not associated with changes in anxiety levels [27]

#### Age

Associations between age and preferred role were reported in 13 different studies. Six studies on various patient populations (women with breast cancer [35,54], patients with lung cancer [53], patients with asthma [40] patients visiting their GP [43] and general public [20] found that older patients preferred more often a passive role in medical decision-making, four studies (three among patients with breast cancer [12,35,44] and one among patients with chronic hepatitis [50] found that younger patients preferred more often an active role, and three studies (all among patients with breast cancer [18,35,54] found that younger patients preferred more often a shared role. Finally, three studies (one among patients with breast cancer [37], one among patients with colorectal cancer [33] and one among patients with different types of cancer [38] found no association between age and preferred role in medical decision-making.

Three studies reported associations between age and perceived role (all among patients with breast cancer) of which one found that older women perceived a more passive role than preferred [37], one found that younger patients perceived a less active role than preferred [49] and one found no association [44].

One study reported no association between age and congruence was found [17].

#### Gender

One study in patients with haemato-oncological illnesses [17] reported no association between gender and congruence between preferred and perceived participation.

#### Education level

Seven studies reported on associations between education and preferred role using different definitions of higher education (for instance ‘high school completed or higher’ [12], ‘bachelor degree’ [26], ‘college degree’ [42]).

Five studies (four among patients with breast cancer [12,26,42,54] and one among the general public [20]) found that higher educated people more often prefer an active role, two studies (one among patients with breast cancer [50] and one among patients with asthma [40]) found that lower educated people more often prefer a passive role. One study among patients with breast cancer reported no association between education and perceived role [35].

Two studies reported on association between education and congruence of which one among patients with breast cancer [49] found that lower educated people preferred less participation than perceived and the other study among patient with solid and haematological cancer [17] did not find an association between education and congruence.

#### Socioeconomic status

One study among the general public [20] reported that patients with a higher income more often preferred an active role.

#### Ethnicity

One study reported that black patients prefer a more passive role compared to white patients [20] and one study reported that Latina-Spanish speaking women with breast cancer [58] preferred more involvement than perceived in the decision-making process compared to Latina-English speaking, African American and Caucasian women with breast cancer.

#### Marital status

An association between marital status and preferred role repoted in three studies. Of these, one study among patients with breast cancer [35] reported that widowed women preferred more often a passive role in medical decision-making than partnered, divorced, separated or never married women, another study among women with breast cancer [12] found that women who were married were more likely to prefer active or collaborative roles in treatment decisions, and one study among patient with HIV/AIDS [36] showed that patients with a partner preferred a less active role than patients not having a partner.

#### Quality of life

Two studies reported no association between quality of life and congruence. One among patients with primary breast cancer [57] and one among patients with advanced lung cancer [52].

#### Depression or anxiety

One study [46] showed that patients with primary breast cancer with a preference for a passive role in decision making were more depressed than patients with a preference for active role in decision making. A sample of patients with breast cancer [57] showed that patients who participated in decision making as much as they preferred had lower depression scores than patients who participated less than preferred.

A study among patients with different types of cancer [27] showed that role mismatch was associated with changes in anxiety before and directly after consultation with their oncologist.

### Interventions

Four randomized controlled trials reported results concerning preferred and perceived role in medical decision making.

One study included patients with multiple sclerosis evaluating an evidence-based patient decision aid on multiple sclerosis immunotherapy [30]. The percentage of congruence between preferred and perceived role during consultation did not differ between the group with decision aid and group with standard information.

A study investigated patient education on prostate cancer screening among men who underwent a health maintenance examination at a family practice, and its influence on involvement in decision making [45]. One group received a web based decision aid, another group received a paper based decision aid and a third (control) group received no previsit education. Patients with either decision aid more often perceived an active role in decision making compared to the control group.

One study evaluated the feasibility of using a computer intervention to enhance communication between health care professionals and women with breast cancer [31]. Women in the intervention group were encouraged to use the information and decision preferences profiles generated by the computer program at their clinic appointment and women in the control group simply completed measures of decision preference before their clinic appointment. A significantly higher number of women in the intervention group reported that their perceived role was more passive than they preferred beforehand.

One study evaluated a cancer consultation preparation package (CCPP) designed to facilitate patient involvement in oncology consultations in men and women with different types of cancer [29]. Patients received either this CCPP with a question prompt sheet, booklet on clinical decisions and patient rights and introduction to the clinic, or a control booklet and introduction to the clinic. Patients that received the CCPP were less likely to receive their preferred role in decision making than the control group.

## Discussion and conclusion

### Discussion

This study reviews the findings of 44 studies (including 52 patient samples) reporting on patients’ preferences for participation in medical decision-making and congruence with perceived participation. Commonly, most patients preferred a shared role in the decision-making process. However, in studies that assessed preferences retrospectively, the majority of patients more often preferred a passive role. Also, patients with cancer more often desired a passive role than non-cancer patients and when preferences were asked in general, patients more often desired a passive role than when asked about more specific treatment option.

We found that the mean of patients whose perceived role in decision-making was congruent with their desired role was 60%. In case of no congruence, patients in general preferred more participation in the decision-making process than perceived. Furthermore, this review showed that in studies in which preference was retrospectively measured congruence was more often found, compared to the prospective studies. Percentages of congruence was similar for patients with cancer and patients without cancer and also regardless of whether preferences related to general or specific treatment options. Lastly, several associations were reported with patient preference for participation in medical decision-making, most commonly age and education level, and most frequently reporting that older people and people with a lower education prefer more often a passive role.

This review gives health care professionals (more) insight in patient wishes and experiences with treatment decision-making and could help them guide patients in treatment decision-making.

### Strengths and limitations

A strength of this review is the broad approach. Most research concentrates on patient participation in cancer decisions, but treatment decision-making is relevant to a much broader range of patients than cancer care alone. Therefore we included all patient populations (e.g. patients with cancer patients and patients with other conditions). Furthermore, in addition to previous reviews, we drew distinctions between prospective and retrospective measurement of preferences and type of treatment decisions (i.e., general or specific).

Although this review covers a substantial number of studies on patient preference for participation in decision-making, it also has limitations. First, studies included in this review were only those in the English language, and although covering most parts of the world, they are conducted in wealthy countries. This limits the generalizability of our findings. Secondly, we only included studies in which congruence was given or could be calculated. Therefore, we did not retrieve all studies relevant to preferences. Third, we recoded the reported data on patient participation on a 5-point scale into three categories (active, shared or passive) before calculating congruence, this may have caused an overestimation of congruence.

### Preferences and congruence with perceived participation

Overall our findings are in accordance with the findings of Tariman and colleagues among patients with cancer [10]. While our study included over 10 studies published subsequent to their review and 8 studies among patients with other conditions: in most studies a majority of people prefer a shared role, in all studies there is a group in which there is a mismatch between preferred and perceived role and in case of this mismatch this is more often in the direction of preferring more participation than perceived. Despite of this overall accordance in findings, our review shows that there seems to be some differences between patients with cancer and patients with other diagnoses. Patients with cancer appeared to more frequently prefer a passive role in participations, while there appeared to be no differences in the occurrence of mismatch. Yet when a mismatch occurred, it concerned patients with cancer who preferred less participation than those with other conditions [18,26,28,29,39,44,49,51],[57]. It should be noted that decisions in cancer care may differ from non-cancer diseases. Ernst et al. [17] argue that preferences for a passive role in haematology patients may be a consequence of stress as a result of the invasive and complex therapies.

While in case of a mismatch generally patients preferred a more active role (in 36 patient samples), it is important not to forget that we found nine patient samples in which more patients with a mismatch preferred a less active role than perceived. Further, in *all* studies there were patients that preferred less participation. For these patients, the idea that generally more participation should be advocated can be questioned. Age and educational level were most frequently associated with preferred level of participation in medical decision-making, finding that older patients and lower educated patients preferred a less active role. Simply following these preferences, might cause inequity in health care provision, as older and lower educated patients are often the most vulnerable people. In order to foster the health of these patients, it might be necessary to promote their participation and empower them to take an active role in decision-making. Studies showed that active participation was associated with positive health outcomes such as overall quality of life, higher physical and social functioning and less fatigue [35]. Yet, one should also be aware of possible negative effects. Some studies showed that pushing patients to play an active role in medical decisions could have negative consequences such as decisional regret [51], increased anxiety [59] or less confidence that they make the right treatment decision and unnecessary stress [18].

### Prospective versus retrospective assessment of preference

We found some differences between the 12 studies in which preferences were measured before the decision-making took place (prospectively) and the 37 studies in which preferences were measured after the decision-making took place (retrospectively). This suggests that there is indeed a fundamental difference between the two ways of studying preferences. One difference we found was that when preference was assessed retrospectively, patients seemed more frequently prefer a passive role in decision-making. This is in line with a study that found that relying on the physicians’ expertise and trust in the physician can play a role in preferences regarding patient participation [60].

Another difference we found is that mismatch less often was found in retrospective studies than in prospective studies. This is not surprising given that in retrospective studies the assessment of preference probably includes an element of evaluation of the decision-making that occurred. This may be due to patients’ desire for congruence, introducing cognitive bias into the response. Yet, this potential desire for congruence did not result in 100% congruence. That mismatch is found in retrospective assessment of preferences in part of the patients suggests that patients are able to separate preference from evaluation or satisfaction with the decision-making at least to some extent. Taking this into account one could argue that retrospective assessment of preference might be as suitable as prospective assessment of preferences. Practically it is easier to measure preferred and perceived participation at the same moment. More substantially, compared to prospective assessment it takes the evolution of preferences into account, since preferences can evolve during the decision-making process, as reported by several authors [15,61]. On the other hand, prospective measurement of course holds the benefit of most accurate assessing preferences.

### Conclusion

This review shows that patients’ preferences for participation in medical decision-making vary. This review also shows that congruence is achieved in about 60% of the cases, and that in cases in of mismatch, patients most often preferred more involvement. Nevertheless, there was also a substantial group of patients that preferred less involvement than perceived. These results hold both for studies that measured preferences prospectively and retrospectively. Yet we did find that there are differences between the two methods of measuring preferences, it appears that the experience of the decision-making influence retrospective measurement. This leads to somewhat higher proportions of people preferring a passive role and more frequent mismatch in retrospective measurement compared to prospective measurement. Although some studies found associations with socio-demographics, patient preference for participation in medical decision-making seems individual. Therefore it is important that physicians discuss the preferred role with their patient in order to fit the decision-making process to the needs and capabilities of the individual patient.

## Competing interests

The authors declare that there is no conflict of interest.

## Authors’ contributions

All authors (LB, WH, DRMT, GAMW, HRWP and BDOP) participated in the conception and design of this review. LB and WH reviewed the titles and abstracts. LB, WH, HRWP and BDOP reviewed the full papers to determine which to include in the review. All authors were involved in the interpretation of the findings. All authors were involved in either drafting the manuscript or providing revisions. All authors read and approved the final manuscript.

## Pre-publication history

The pre-publication history for this paper can be accessed here:

http://www.biomedcentral.com/1472-6947/14/25/prepub
